# Road-Adaptive Precise Path Tracking Based on Reinforcement Learning Method

**DOI:** 10.3390/s25154533

**Published:** 2025-07-22

**Authors:** Bingheng Han, Jinhong Sun

**Affiliations:** 1 School of Information Science and Engineering, Fudan University, Shanghai 200433, China; 2 Department of Electrical and Electronic Engineering, The Hong Kong Polytechnic University, Kowloon 999077, Hong Kong

**Keywords:** soft actor–critic, pure pursuit control, path tracking, motor efficiency map

## Abstract

This paper proposes a speed-adaptive autonomous driving path-tracking framework based on the soft actor–critic (SAC) and pure pursuit (PP) methods, named the SACPP controller. The framework first analyzes the obstacles around the vehicle and plans an obstacle-free reference path with the minimum curvature using the hybrid A* algorithm. Next, based on the generated reference path, the current state of the vehicle, and the vehicle motor energy efficiency diagram, the optimal speed is calculated in real time, and the vehicle dynamics preview point at the future moment—specifically, the look-ahead distance—is predicted. This process relies on the learning of the SAC network structure. Finally, PP is used to generate the front wheel angle control value by combining the current speed and the predicted preview point. In the second layer, we carefully designed the evaluation function in the tracking process based on the uncertainties and performance requirements that may occur during vehicle driving. This design ensures that the autonomous vehicle can not only quickly and accurately track the path, but also effectively avoid surrounding obstacles, while keeping the motor running in the high-efficiency range, thereby reducing energy loss. In addition, since the entire framework uses a lightweight network structure and a geometry-based method to generate the front wheel angle, the computational load is significantly reduced, and computing resources are saved. The actual running results on the i7 CPU show that the control cycle of the control framework exceeds 100 Hz.

## 1. Introduction

The rapid development of autonomous driving is due to advances in many fields, including artificial intelligence, sensor technology, computer vision, and control theory [1]. Achieving safe and efficient vehicle driving has become a research focus. In this field, path planning, speed optimization, and path tracking are the three key components for achieving safe autonomous driving.

Effective path planning that considers safety, driving efficiency, passenger comfort, and energy consumption can ensure that vehicles drive safely in complex environments and avoid potential collision risks. To realize this, researchers have proposed a variety of path-planning methods: graph search, sampling, model-based algorithms, etc. The work mentioned in [2] improved the artificial potential field algorithm to solve a series of problems in path planning, such as unreachable targets, local minima, and adaptation to dynamic obstacles. At the same time, they designed an improved sliding mode controller to verify the feasibility of the path and tested it at different speeds. The results showed small lateral and heading errors and maintained high tracking accuracy. However, there are some limitations in their research, such as the use of a two-degree-of-freedom vehicle model, the failure to consider external factors such as road conditions and weather, slow calculation speed, and limitations to simulation experiments. Paper [3] proposed a path smoothing algorithm based on an improved RRT*, which generated long-distance paths based on an adaptive node expansion model and effectively improved the convergence speed, obstacle avoidance ability, and planning efficiency. However, this method required expanding a large number of nodes and evaluating these nodes to adjust their weights. It led to the existence of redundant nodes, that need to be removed by combining a filtering algorithm, thereby increasing the complexity of the algorithm. In addition, this method was mainly applicable to low-speed scenarios. Z. Bai et al. proposed a new comprehensive reward function [4], which aimed to enable the robot to reach the target location quickly and safely. In addition, they designed an action evaluation system that effectively balances the relationship between exploration and utilization, and the system adapted to different outdoor environments. However, it mainly focused on testing static scenes, which requires maintaining a relatively complex grid map. In addition, it did not analyze or verify the disturbances caused by changes in its mass. The authors in Ding [5] used a vehicle dynamics model predictive control algorithm to model the vehicle and optimize the generated obstacle avoidance path to meet the constraints of vehicle dynamics while improving driving safety. However, it failed to take into account the disturbance and energy of the vehicle during driving.

Based on the path-planning module, further refining the vehicle’s driving speed by optimizing the vehicle speed and acceleration can significantly improve driving efficiency and comfort [6]. In addition, by using the motor energy efficiency map, the speed of the autonomous driving vehicle can be optimized, thereby maximizing energy efficiency while ensuring driver safety and comfort. Y. Zhang et al. proposed an optimized energy-saving control scheme that takes into account driving scenarios and multiple energy-saving constraints in [7]. The controller combined the motor energy efficiency map to find and optimize the motor output efficiency corresponding to the speed at each moment. However, this scheme did not fully optimize the reference path of the system, so it could not fully reflect the driving preferences of human drivers. In [8], a decision framework for autonomous vehicles based on safety and energy saving was constructed. This framework effectively avoided rear-end collisions during autonomous driving and improved energy-saving efficiency. However, this framework did not optimize the energy utilization efficiency of autonomous vehicles throughout the process, making it difficult to ensure that the motor operates stably within the expected energy area. A path planning and trajectory generation algorithm based on a minimum time that takes into account the robot’s speed, acceleration, and jerk constraints was proposed in [9]. The algorithm was optimized using the artificial bee colony nature-inspired optimization algorithm. The final generated trajectory satisfies the robot’s dynamic constraints. However, this article did not consider the internal and external disturbances that exist during the robot’s driving process.

Path tracking requires the real-time acquisition of the vehicle’s state information in real time and compare it with the reference path to ensure that the vehicle can accurately drive along the target path [10]. The geometry-based PP method was proposed by Craig Coulter in 1992, it is simple to control, and does not require complex kinematic and dynamic models. Paper [11] used the backward Euler method to improve the lateral error accuracy during vehicle tracking, optimize the acceleration and steering angle of the control input, enhance the real-time processing tracking performance, and increase the controller’s stickiness, along with employing hot start technology to accelerate convergence. The shortcomings included the heading error becomes larger, the amount of calculation increases, and the internal and external disturbances in the control process are not fully considered. A vehicle following model considering a time safety strategy was proposed in [12]. This scheme also analyzed the relationship between vehicles in different time periods during the following process, improving the following efficiency and safety performance. However, it failed to consider the impact of different road surfaces on vehicle following, and also lacks consideration of the energy consumption during the following process. The method presented in [13] integrated behavioral decision making, path planning, and motion control into a unified module for autonomous driving on highways. However, this study mainly focused on decision making and path generation, and did not consider the internal and external disturbances that may occur during driving. In addition, no effective solution was proposed in this paper to solve the deviation between simulation and reality. An adaptive tracking controller based on reinforcement learning (RL) is proposed in [14], which enhances the stability of the network through model previewing and adaptive correction. However, the paper does not discuss the computational complexity in detail, nor does it consider the potential disturbances that may occur during the tracking process. Paper [15] proposed a reinforcement learning (RL) controller framework that could directly generate steering angles and accelerations without requiring an understanding of the dynamic and kinematic models of the vehicle. However, the stability of the vehicle during driving needs further analysis, and how to narrow the gap between simulation and reality also needs to be discussed. The work in the paper [16] directly used images as input to generate vehicle control instructions based on an RL framework, thereby reducing the intermediate links and avoiding dependence on vehicle kinematic and dynamic modeling. However, this solution mainly focused on learning the vehicle driving strategy and did not consider the various disturbances that may occur during driving. The vision-language model-RL proposed in [17] combined a pre-trained vision-language model with reinforcement learning, offering a new method for generating reward signals that overcomes the limitations of traditional RL approaches. In paper [18], an event-triggered reinforcement learning model predictive control (MPC) path tracking control system was proposed, which effectively reduced the computation time. However, this study did not consider the possible disturbances that may occur during the tracking process, and also lacked a sufficient analysis of the energy consumption during driving. MPC utilized the dynamic model of the vehicle to achieve path tracking by optimizing the control input which can handle multiple constraints and is suitable for dynamic environments. The SAC algorithm is an efficient and stable policy-based RL method suitable for continuous control tasks. Combining the motor energy efficiency map with SAC enables intelligent acceleration and speed decisions during path tracking, improving energy efficiency, safety, and comfort.

This paper pays special attention to the uncertainty and performance requirements of the vehicle during driving and designs targeted evaluation functions to ensure that the autonomous vehicle can not only quickly and accurately track the path during path tracking, but also effectively avoid obstacles and keep the motor running in the high-efficiency range, thereby reducing energy loss. It is worth mentioning that the entire framework adopts a lightweight network structure and a geometry-based front wheel angle generation method, which greatly reduces the amount of calculation and saves computing resources. In actual tests, the results obtained using the i7 CPU show that the control cycle of the framework exceeds 100 Hz, verifying its efficiency and practicality. The CarMaker tool and the Logitech G29 Driving Force steering wheel and pedals constitute the test platform. The main application scenario of this design is urban roads, and CarMaker uses a single model.

Main contributions and novelties can be summarized as follows:

- By utilizing the motor energy efficiency map, the energy-efficient output control strategy based on the SAC method ensures that the motor of the autonomous vehicle operates in an efficient state at all times. This approach not only saves energy but also prolongs the service life of the motor.
- By adopting U-Net and pavement image segmentation methods, we developed a real-time detection system for road surfaces to better address the complexities of road conditions. By incorporating the results into the soft actor–critic (SAC) controller, it can effectively handle external disturbances caused by changes in the friction coefficient, thereby enhancing its stability.
- Our work directly uses the results of road segmentation to determine the category to which each pixel belongs (such as normal road, unpaved road, etc.) by performing semantic segmentation on the input image. The segmented pixel information is then input into the SAC network. Unlike directly inputting image features into the SAC network, this method enables the SAC network to obtain the road type of each pixel, thereby enhancing its adaptability.
- The SAC controller can better represent the vehicle’s dynamic model and make future predictions based on the reference path, thereby improving the ability to handle disturbances during tracking.
- The SACPP controller only needs to obtain the nominal values of the vehicle parameters without requiring the actual values. In addition, due to its simplified network structure and geometric control method, the amount of calculation is significantly reduced, thereby improving the practicality of this algorithm.

Section 2 introduces the problem and its framework. Section 3 shows how to generate a smooth reference path using the improved A* algorithm and the conjugate gradient descent method. Section 4 designs a robust controller that combines vision processing and with SAC algorithm to address road surface uncertainty. Section 5 develops a PP-based path-tracking controller. Finally, Section 6 verifies the proposed method through simulation and successfully tests it on an actual vehicle.

## 2. Problem Formulation

**Figure 1:**
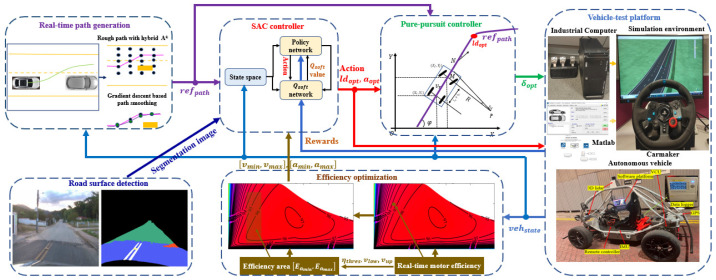
The framework of the whole system.

Our work proposes a speed-adaptive autonomous driving path-tracking framework based on the SAC method, as shown in Figure 1. This framework is constructed with six modules: real-time path generation, road surface detection, efficiency optimization, SAC controller, pure-pursuit controller, and the vehicle-test platform. The vehicle state ${veh}_{state}$ includes the vehicle center coordinates (x,y), heading angle φ, vehicle speed *v*, acceleration *a*, and front wheel angle δ.

1. In the real-time path generation module, the hybrid A* method [19] is used to generate and avoid obstacles according to the generated cost function to generate a safe driving path while avoiding obstacles. However, in complex environments, this path may have some uneven parts. Thus, a gradient descent smoother is adopted to optimize the path, focusing on the curvature and smoothness to improve the overall driving experience and provide the reference path ${ref}_{path}$.
2. The vision-based road semantic segmentation algorithm and U-Net network structure are used to segment the state of the road surface in real time during driving [20,21], as shown in the road surface detection module. It can directly semantically segment different types of roads, each corresponding to a specific category and indirectly reflecting different friction coefficients. These classification results will be input into the RL model as feature values, thereby enhancing its perception of the external environment.
3. The vehicle’s driving stability, safety, and motor output efficiency are always considered to ensure that the motor operates within the efficient output range, as demonstrated in the efficiency optimization module. First, a motor energy efficiency model is built to describe the energy consumption at different speeds $\left[v_{min},\;v_{max}\right]$ and accelerations $\left[a_{min},\;a_{max}\right]$. Then, we include energy efficiency $\left[E_{a_{min}},\;E_{a_{max}}\right]$ as part of the reward function to encourage the algorithm to choose a more efficient driving speed.
4. Based on input information such as ${ref}_{path}$, semantic information on road segmentation, ${veh}_{state}$, and $\left[E_{a_{min}},\;E_{a_{max}}\right]$, SAC controller can adjust the vehicle’s optimal acceleration $a_{opt}$ and forward distance ${ld}_{opt}$ in real-time, as shown in the SAC controller module.
5. The PP geometric tracking method used in the pure-pursuit controller is used to reduce the amount of algorithm calculation. $a_{opt}$ and ${ld}_{opt}$ are directly used to calculate the optimal front wheel angle $\delta_{opt}$. It has almost negligible calculation time while meeting the tracking accuracy, which significantly improves practicality.
6. As demonstrated in the vehicle-test platform, the simulation platform is mainly composed of an industrial computer, the CarMaker, and a Logitech steering wheel and brake throttle system. The platform is mainly used for data collection and network training. Additionally, the actual vehicle is used to finally verify the performance of our work.

## 3. Real-Time Path Generation

Under the premise of fully considering energy savings, we used the path planner developed in [22] to generate a preliminary path $\left\{r_{1},\;r_{2},\ldots ,r_{n}\right\}$. Although this path is feasible, unnecessary steering actions often occur, making it beneficial to post-smooth the path to improve comfort and safety. Thus, a gradient descent smoother is proposed to minimize the cost function *J* of the path. To calculate the gradient of each objective function, we need to take partial derivatives of them. We set the coordinates of the vertices on the path to $R_{i}=\left(x_{i},y_{i}\right)$, where $r_{i}\;\left(1<i<n\right)$.

(1) $$J_{cur}=\lambda_{cur}\sum_{i=1}^{n-1}{\left(\left(R_{i}-R_{i-1}\right)-\left(R_{i+1}-R_{i}\right)\right)}^{2}$$

The cost function $J_{cur}$ given in (1) evaluates the curvature of the path by calculating the change in angle between adjacent vertices. The weight coefficient $\lambda_{cur}$ represents the adjustment parameter of the curvature cost function, which is primarily used to optimize curvature, allowing the evaluation results to better meet actual needs. This term aims to minimize the change in curvature of the path, thereby ensuring both the smoothness and feasibility of the path. In addition, the degree of path change is controlled by the curvature weight $J_{cur}$.

(2) $$J_{smo}=\lambda_{smo}\sum_{i=1}^{n-1}{\left(\Delta R_{i+1}-\Delta R_{i}\right)}^{2}$$

The cost function $J_{smo}$ in (2) is obtained by calculating the displacement vectors between the adjacent points. It assigns costs to unevenly spaced vertices and adjusts their directions to ensure the continuity and smoothness of the path. The path-smoothing weight $\lambda_{smo}$ is used to influence changes in the path. Specifically, the displacement vectors are defined as $\Delta R_{i}+1=R_{i}+1-R_{i}$ and $\Delta R_{i}=R_{i}-R_{i}-1$. Thus, our objective function can be expressed in (3):

(3) $$J=J_{cur}+J_{smo}$$

We start with an initial path, calculate the gradient at each point, and then search in the direction of the negative gradient. This process is repeated until the objective function converges or the maximum number of iterations is reached. In each iteration, we set a convergence condition and stop the search when the condition is met, otherwise we continue to iterate. In this way, we can gradually optimize the path and eventually obtain a smooth path that meets the vehicle’s kinematic constraints. The specific steps are as follows:

1. Initialize the waypoint to the original waypoint.
2. Compute the gradient for each path point $R_{i}$, and calculate the objective function with respect to the gradient of $R_{i}$ gradient in (4):
  (4) $$\nabla J\left(R_{i}\right){=\lambda }_{cur}\nabla J_{cur}+\lambda_{smo}\nabla J_{smo}$$
  Curvature gradient:
  $$\nabla J_{smo}=\;2\left(R_{i-1}-2R_{i}+R_{i+1}\right)$$
  Smooth gradient:
  $$\nabla J_{smo}=\;2\Delta R_{i+1}-\Delta R_{i}$$
3. Waypoint update equation is given in (5):
  (5) $$R_{i+1}=R_{i}-\eta \nabla J\left(R_{i}\right)$$
  where η is the learning rate, which controls the update step size. It is crucial to choose an appropriate η, as a learning rate that is too large may cause algorithm instability, while one that is too small will slow down the convergence speed. Adjust the two weights $\lambda_{cur}$ and $\lambda_{smo}$ according to the specific scenario to achieve the ideal path-smoothing effect and path fidelity. The parameters η selected in this paper are 0.001, $\lambda_{cur}$ and $\lambda_{smo}$ are set to 0.6 and 0.4, respectively. In each iteration, the algorithm calculates the gradient of the objective function and updates the positions of the path points using the conjugate gradient method [23]. Finally, the smooth path will be output as the navigation reference path.

## 4. Optimal Speed Generation

### 4.1. Road Surface Detection

**Figure 2:**
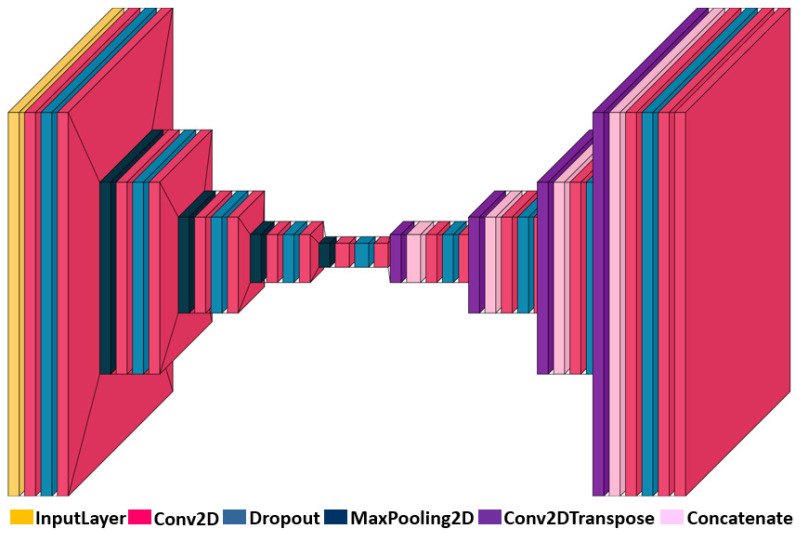
Summary of the road types.

As demonstrated in Figure 2, the road surface detection module uses the U-Net network [24] to capture the contextual information in the image and divide the road surface into 13 different categories: (black, background), (light blue, road asphalt), (greenish blue, paved road), (peach/light orange, unpaved road), (white, road marking), (pink, speed bump), (yellow, cats eye), (purple, storm drain), (cyan, manhole cover), (dark blue, patches), (dark red, water puddle), (red, pothole), (orange, cracks). These features are then incorporated into the SAC state representation. We simplified the model as shown in Table 1, by removing the redundant Dropout layer and merged some convolution operations. This adjustment not only reduces the complexity of the model but also effectively maintains its segmentation ability.

### 4.2. Motor Efficiency Optimization

**Figure 3:**
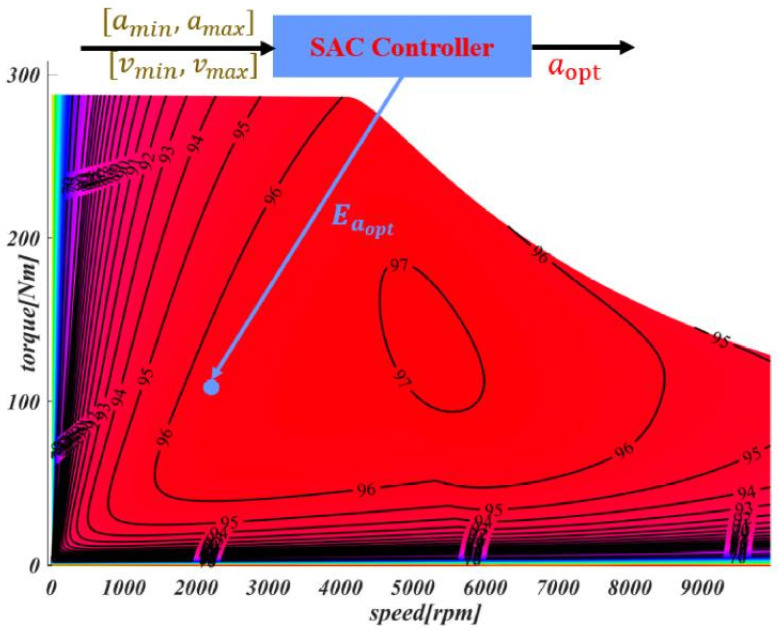
Generation of $E_{motor}$.

As mentioned in the efficiency optimization module and the real-time output of motor efficiency $E_{motor}$, we select the range with a higher output efficiency than the current or set threshold within the converted restriction range. Then, these selections can be converted into $\left[v_{min},v_{max}\right]$ and $\left[a_{min},a_{max}\right]$ of the vehicle. These generated ranges meet the requirements of being higher than the current or set output efficiency $\left[E_{a_{min}},E_{a_{max}}\right]$, while also satisfying the dynamic and kinematic constraints of the vehicle, thereby improving the motor output efficiency and reduces the energy consumption without affecting the vehicle’s driving. Directly applying the acceleration information from the motor energy efficiency map to the SAC controller can effectively optimize energy efficiency and control the performance of electric vehicles. As mentioned in Figure 1 and our previous work [25], the optimal motor efficiency $E_{a_{opt}}$ can be calculated and is demonstrated in Figure 3.

### 4.3. SAC Controller

The SAC controller can be efficiently generated and optimized for speed, as detailed below:

***State***: It should contain various information related to the movement of the vehicle, including vehicle status: position, speed, acceleration, and direction; environmental information: characteristics obtained through the camera, such as pavement with dimensions of 256 × 256; $E_{a_{opt}}$ when generating optimal speed, acceleration, and ${ref}_{path}$. According to the environment and task requirements, the path generated by the hybrid A* is optimized and input into the SAC using the conjugate gradient descent smoother: (${ref}_{x0},{ref}_{y0},{ref}_{x1},{ref}_{y1},\ldots ,\ldots ,{ref}_{x7},{ref}_{y7}$).

***Action***: SAC outputs the optimal acceleration $a_{opt}$ and the optimal look-ahead distance ${ld}_{opt}$ through learning strategies, allowing the vehicle to drive smoothly in complex environments. Thus, the pure-pursuit controller can achieve stable tracking only by updating the optimal front wheel angle $\delta_{opt}$.

The output action is defined by two continuous variables ld, *a*, where *a* is limited to the range [−4, 4] based on the vehicle dynamics. The real-time ld is based on the vehicle’s dynamic constraints and is limited to the range $\left[0,\;ld_{max}\right]$.

***Reward***: The reward function is designed to encourage vehicles to minimize energy consumption and drive more efficiently while ensuring tracking accuracy, safety, and comfort.

1. Path tracking error: The tracking error includes the lateral tracking error (cross-track error, (cte)) and the heading tracking error eψ. During the tracking process, as long as the tracking error of the controller is kept within a reasonable range, the safety of driving can be ensured. Therefore, the reward function will encourage the controller to minimize both the lateral and heading errors, especially the lateral error.
  $$r_{cte}=\left\{\begin{array}{c} \xi_{1}*\left(\frac{w_{veh}}{2}-cte\right)\;\;\;\;cte<\frac{w_{veh}}{2}\\ -r_{max}\;\;\;\;\;\;\;\;\;\;\;\;\;\;\;\;cte\geq \frac{w_{veh}}{2} \end{array}\right.$$
  $$r_{e\psi }=\left\{\begin{array}{c} e^{-\xi_{2}\left|e\psi \right|\;\;\;\;\;\left|e\psi \right|<\frac{\pi }{2}}\\ -r_{max}\;\;\;\;\;\left|e\psi \right|\geq \frac{\pi }{2} \end{array}\right.$$
  Since the heading deviation has a significant impact on driving safety, the reward function will decrease exponentially as eψ increases. This design is intended to encourage the controller to pay more attention to the heading error to ensure driving safety.
2. Velocity tracking error: In terms of tracking efficiency and comfort, we have established a reward mechanism: within the speed limit of the road, the faster the speed, the higher the efficiency. Therefore, we introduced a speed reward. When the vehicle speed is lower than a certain set value, a negative reward will be given to ensure that the vehicle can maintain a certain initial speed.
  $$r_{v}=\left\{\begin{array}{c} \xi_{3}*\left(v-v_{min}\right)\;\;\;\;\;\;\;v>v_{min}\;\\ -r_{max}\;\;\;\;\;\;\;\;\;\;\;\;\;vs.\leq v_{min} \end{array}\right.$$
  The acceleration of the vehicle directly affects ride comfort, so we have also established an acceleration reward mechanism. Specifically, the smaller the acceleration, the smoother the driving process, thus improving the ride comfort experience.
  $$r_{a}=\left\{\begin{array}{c} \xi_{4}*\left(a_{max}-a\right)\;\;\;\;\;\;a<a_{max}\;\\ -r_{max}\;\;\;\;\;\;\;\;\;\;\;\;a\geq a_{max} \end{array}\right.$$
3. Energy efficiency: Energy consumption is a crucial factor in high-speed path tracking. The motor running at a low energy efficiency point will directly lead to a shortened actual mileage of the vehicle and also shorten the service life of the battery and motor. Therefore, it is very necessary to keep the motor output at a high-efficiency point during driving. As given in the efficiency optimization module, $\left[E_{a_{min}},\;E_{a_{max}}\right]$ can be defined. When $E_{motor}$ exceeds this threshold, the corresponding reward will be given; if it is lower than the set threshold, the reward will be negative.
  $$r_{E_{map}}=\left\{\begin{array}{c} \xi_{5}*\left(E_{motor}-E_{a_{min}}\right)\;\;E_{motor}>E_{a_{min}}\;\\ -r_{max}\;\;\;\;\;\;\;\;\;\;\;\;\;E_{motor}<E_{a_{min}} \end{array}\right.$$

In our study, the hyperparameter tuning process for the SAC algorithm followed a systematic approach to ensure the reproducibility of the results. We selected a set of initial hyperparameters, including the learning rate, discount factor, target network update frequency, and others. The details are presented in Table 2.

### 4.4. Network

**Figure 4:**
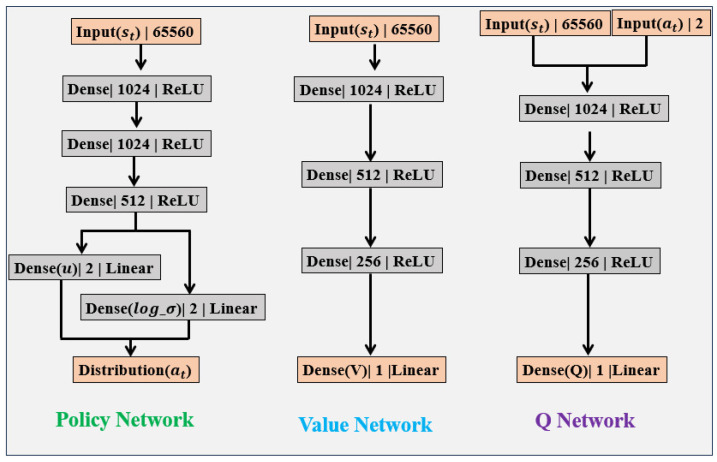
Structure of the SAC network.

In Figure 4, “Linear” refers to, direct linear output without on activation function, while “Dense” means fully connected output. SAC’s experience replay mechanism uses historical data from the cache for learning. For example, 65,560 is calculated as the sum of the segmented image input: 256×256 and the vehicle state: 24. As uniform random sampling may lead to low learning efficiency and even cause overfitting problems, prioritized experience replay is adopted in Algorithm 1 [26]. It samples from the experience replay pool according to a specific priority. Compared with random uniform sampling, it can significantly improve learning efficiency and thus accelerate the convergence process of the model.
**Algorithm 1:** SAC path-tracking training method with prioritized experience replay
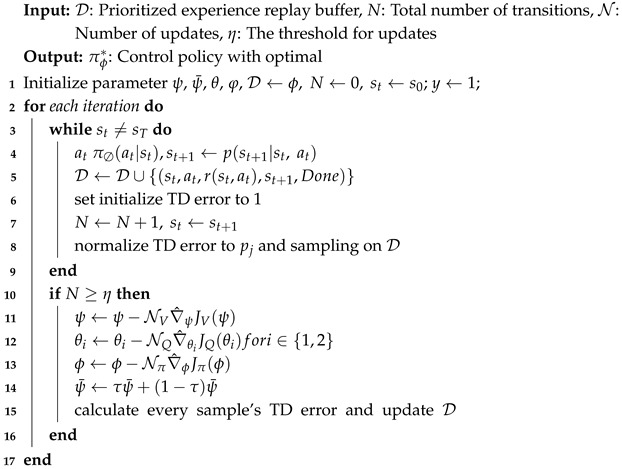


## 5. Path Tracking Using the PP Method

Since MPC has a large computational load, we introduce a pure geometric method to reduce the computational burden while maintaining a tracking accuracy similar to or even better than MPC. In addition, the MPC method also requires more accurate vehicle model data and parameters, which are often difficult to obtain in practical applications.

**Figure 5:**
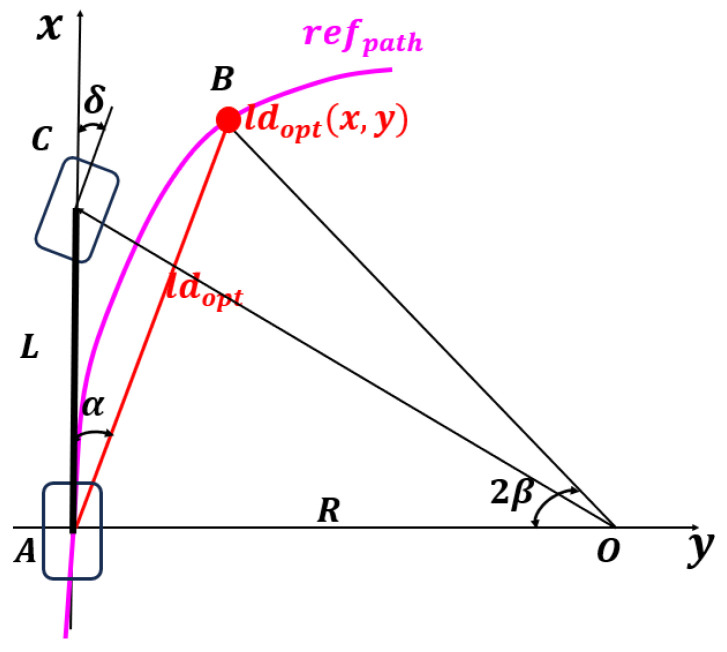
The schematic diagram of PP method.

In Figure 5, based on the current position of the rear axle center of the vehicle, a preview point (x,y) is matched forward on ${ref}_{path}$, recorded as ${ld}_{opt}\left(x,y\right)$, and this preview point is regarded as the goal point for this cycle. Assume that the rear axle center of the vehicle can reach the preview point according to a certain turning radius R. Next, δ is determined based on the geometric relationship between ${ld}_{opt}$, R, and the orientation angle β of the preview point in the vehicle coordinate system. For the center point of the rear axle of the vehicle to smoothly reach point *C* along the arc dotted line path, (6) needs to be satisfied in the triangle OAB:

(6) $$\frac{{ld}_{opt}}{sin2\beta }=\frac{R}{\sin(\frac{\pi }{2}-\beta )}$$

As sin2β=2sinβcosβ, $\sin\left(\frac{\pi }{2}-\beta \right)=cos\beta$, so the above formula can be simplified to (7)

(7) $$R=\frac{{ld}_{opt}}{2sin\beta }$$

In the Ackermann turn (triangle OAC), we have (8)

(8) $$tan\delta \approx \frac{L}{R}$$

Combining the above formulas, we can obtain (9):

(9) $$\delta =\tan^{-1}\left(\frac{2Lsin\beta }{{ld}_{opt}}\right)$$

The lateral position error is defined as the lateral error between the vehicle’s current posture and the forward-looking angle, that is, $e_{y}={ld}_{opt}sin\alpha$. Therefore, (10) is given:

(10) $$\delta =\tan^{-1}\left(\frac{2Le_{y}}{{{ld}_{opt}}^{2}}\right)$$

Since the front wheel turning angle is generally small, the small angle assumption can be used, leading to (11).

(11) $$\delta \approx \frac{2Le_{y}}{{{ld}_{opt}}^{2}}$$

## 6. Experiments and Results

The main parameters of the vehicle and its motors are given in Table 3.

### 6.1. SACPP Network Training

**Figure 6:**
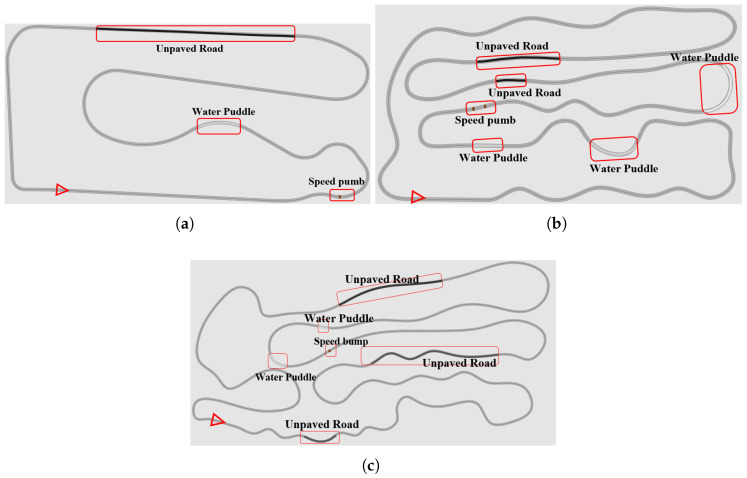
Maps utilized in this work: (**a**) Training scenario 4. (**b**) Training scenario 5. (**c**) Training scenario 6.

In Figure 6, the test scenario built in CarMaker [27] introduces a variety of turning radii and different types of road surfaces while adjusting the mass of the vehicle during the test to evaluate the controller’s tracking performance and anti-interference ability.

1. Training scenario 1: Straight driving on a normal road, 1.87 km long, mainly used to learn basic acceleration and deceleration operations.
2. Training scenario 2: Circular driving on a normal road, 6.366 km long, aimed at mastering basic left and right turn operations.
3. Training scenario 3: Normal road with a combination of straight lines and arcs, 3.61 km long, aimed at achieving simple straight driving, acceleration, and deceleration, and basic operations such as left and right turns.
4. Training scenario 4: A simple scenario with different road surfaces, combining straight lines, arcs, and U-turns, 4.02 km long. This scenario helps the controller adapt to disturbances caused by road changes.
5. Training scenario 5: A complex scenario with a variety of road types, including straight lines, arcs, continuous turns, and multiple U-turns, with a length of 5.184 km. Since this scenario involves different road types, continuous turns, and diverse U-turns, it aims to improve the controller’s precise tracking capabilities in complex environments.
6. Verification scenario 6: This is a complex test scenario (straight line + arc + continuous turns + multiple U-turns) with a length of 7.787 km, covering a variety of road types, continuous curves, and multiple U-turns. This scenario is designed to fully verify the performance of the algorithm in actual applications.

### 6.2. Generalization Performance Verification

**Figure 7:**
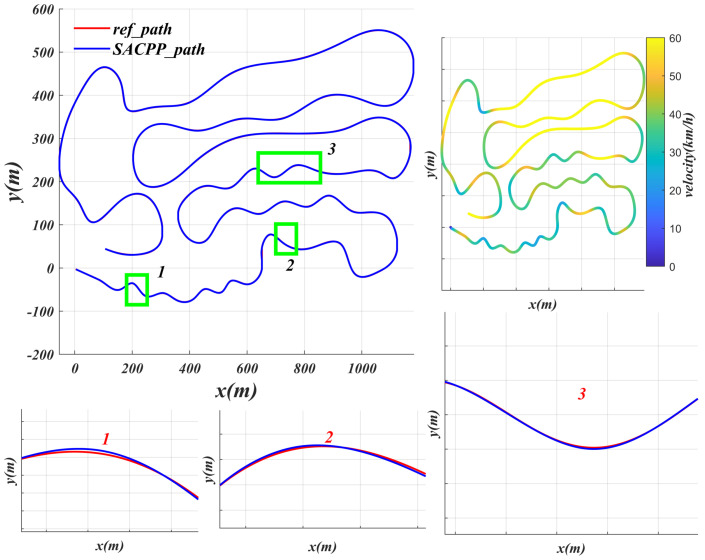
Faced with the complex road conditions of scenario 6, the performance of the SACPP controller in an urban environment is evaluated: red represents the reference path, blue indicates the path of the vehicle under the SACPP controller, and the highlighted paths (1, 2, and 3) are partially enlarged for comparison.

65 km/h This work is mainly aimed at urban environments, where the maximum *v* is 65 km/h. In Figure 7, the controller can reach 65 km/h and the average *v* is 42.3 km/h in a complex environment with continuous turns, U-turns, and different road conditions. The controller SACPP will decelerate in advance when turning, especially on curves with larger arcs, and will implement more significant deceleration measures to ensure that the vehicle has sufficient support while turning, maintaining good tracking ability and ensure driving safety.

**Figure 8:**
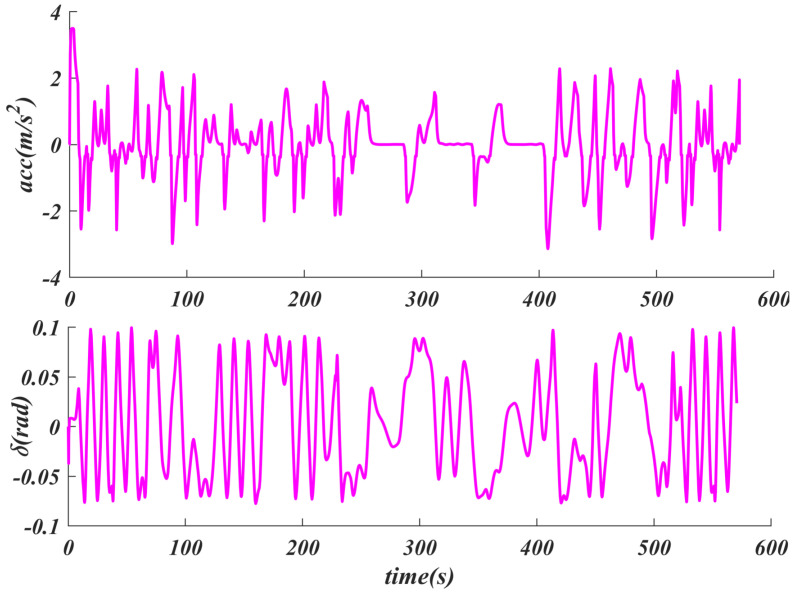
Performance analysis of SACPP controller: acceleration/ deceleration *a*, and steering angle δ constraints in urban driving conditions.

As shown in Figure 8, the maximum acceleration is less than $4\;m/s^{2}$, which meets the vehicle’s dynamic constraints. At the same time, the maximum deceleration does not exceed $-4\;m/s^{2}$, which is close to the actual mechanical limit of the vehicle. The SACPP controller can make full use of the physical characteristics of the vehicle and the road speed limit constraints to generate efficient control variables, maximize the tracking speed under multiple constraints, maintain tracking accuracy, and improve driving safety. Furthermore, the maximum value of δ is close to 0.1rad (about 5.73 degrees), which is much lower than the maximum steering angle of the vehicle. This indicates a smaller turning amplitude, especially when driving at high speeds; a smaller turning amplitude will help improve the stability of the vehicle.

### 6.3. Uncertainties

**Figure 9:**
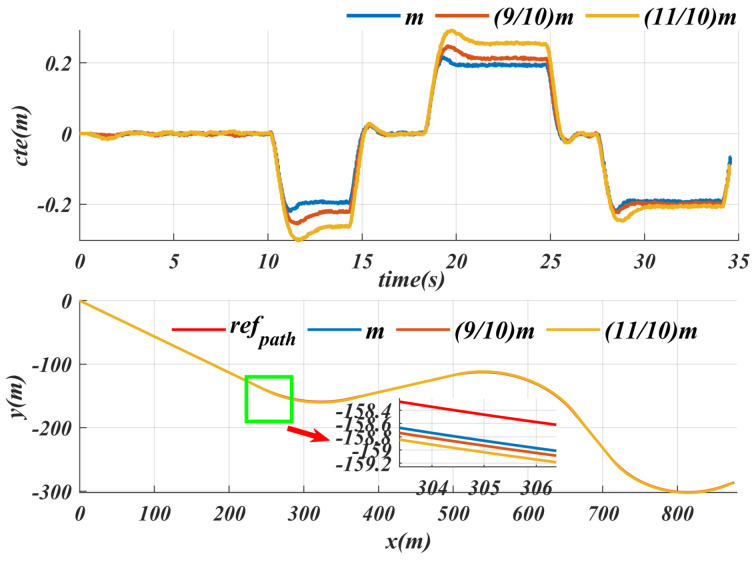
The impact of vehicle weight variations (m, 9/10 m, 11/10 m) on controller path-following performance: a detailed presentation of the average and maximum lateral errors for a given path.

Since changes in vehicle weight introduce external disturbances, we conducted path tracking tests with different masses on the same given path to verify the generalization performance of this algorithm. Figure 9 shows that, for the nominal weight, the average lateral error (cte) is 0.0989 and the maximum cte is 0.219; when the weight is reduced by 10%, the average cte is 0.108 and the maximum cte is 0.254; and when the weight is increased by 10%, the average cte is 0.123 and the maximum cte is 0.302. From the above data, it can be seen that, when the mass change is around 10%, the maximum cte in all cases is less than 0.5. However, compared to reducing weight, increasing weight has a more significant impact on the controller, with the maximum cte exceeding 40% of nominal weight and the average cte exceeding 24% of normal weight. In contrast, when reducing weight, both the maximum and average cte are close to normal weight, indicating that the disturbance caused by increasing weight is much greater than that the disturbance caused by reducing weight.

**Figure 10:**
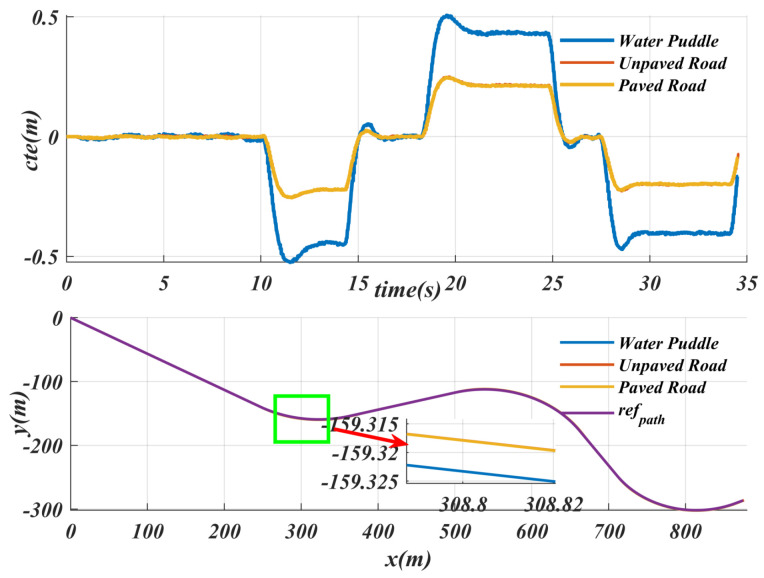
Path-tracking performance of the SACPP controller for a given path under different road conditions: lateral error display for water puddle, unpaved road and paved road.

The path tracking performance under different road conditions was tested, while keeping the same speed for the experiment, and the specific results are shown in Figure 10. For normal paved roads and unpaved roads, the tracking performance is similar, and the maximum cte does not exceed 0.3. However, on slippery roads, the maximum cte is close to 0.5. Although the cte on slippery roads increases at the same speed, it remains within an acceptable range. Road surface detection errors are mainly influenced by lighting conditions, such as incorrectly segmenting paved roads as unpaved roads or failing to accurately identify scenes like puddles. In response to these segmentation errors, this paper pays special attention to the puddle scenario, which refers to relatively slippery road surfaces. The proposed algorithm can ensure that the maximum cte does not exceed 0.5 m. This means that even in the presence of semantic segmentation errors, the algorithm introduced in this paper can still keep the maximum cte within an acceptable range.

From the summary in Table 4, despite the increase or decrease in weight, the maximum cte remains within a reasonable range, which proves that the algorithm has a good handling ability for internal disturbances. It is worth noting that the adaptive speed generation strategy based on SAC can decelerate in advance in slippery road scenarios, thereby reducing cte and ensuring the stability of the driving process. This shows that the algorithm has good adaptability and stability under different road conditions.

**Figure 11:**
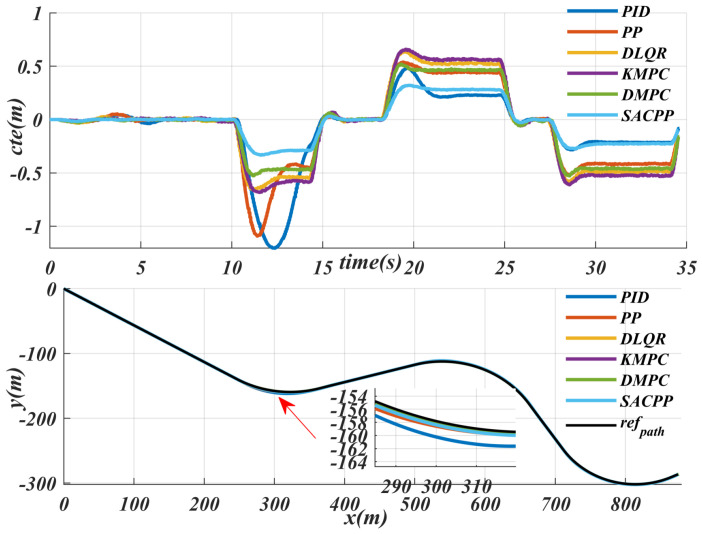
Experimental comparison of internal disturbances caused by robot mass: performance analysis of various control algorithms: such as MPC, DMPC, DLQR, simple geometric tracking and PID.

We conducted an experimental comparison on the internal disturbance introduced by the vehicle mass. The compared algorithms include the model predictive control algorithm (KMPC) based on the dynamic model, the model predictive algorithm (DMPC) based on the kinematic model, the linear quadratic controller algorithm (DLQR) based on the kinematic model, the simple geometric tracking algorithm, and the PID control algorithm. From the comparison results in Figure 11, it can be seen that the MPC controller based on the dynamic model needs to incorporate the vehicle body mass in the modeling process, making it the most affected by the mass. Its maximum cte increases significantly, exceeding 0.5 m, which will seriously affect the safety of the vehicle during driving. For the two control schemes of DMPC and KMPC, their tracking performance is similar, but their maximum cte also exceeds 0.5 m. The tracking performance of the SACPP controller proposed by us remains stable in the face of the uncertainty of mass increase, with little change, and its maximum cte is still within 0.25 m, which greatly ensures the safety of the vehicle during driving.

### 6.4. Energy Consumption

**Figure 12:**
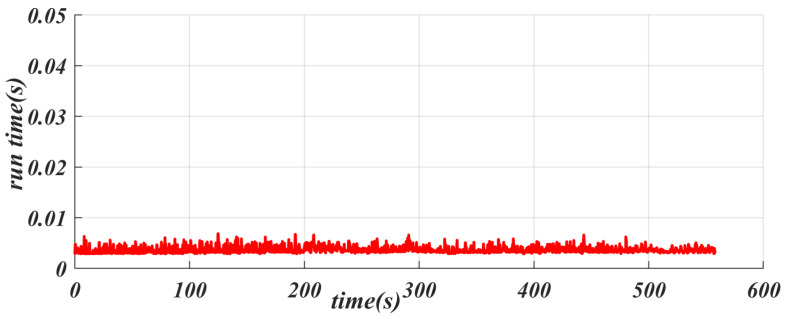
Performance metrics of the proposed SACPP controller: timing analysis and resource utilization on Intel Core i7-9750H and GeForce GTX 1060.

The computer configuration used in the test platform is an Intel(R) Core(TM) i7-9750H CPU, with a main frequency of 2.60 GHz (the actual frequency is 2.59 GHz), and a graphics card of GeForce GTX 1060. During the actual operation process, we measured the algorithm’s time consumption for the proposed controller, and the relevant data are shown in Figure 12. The control scheme proposed here has a maximum computation time of less than 0.01 s and the average time consumed by the algorithm is 0.0037 s; that is, the control cycle exceeds 100 Hz, thus meeting the real-time requirements for path tracking control in autonomous driving. In addition, since SACPP can generate adaptive speed, this feature effectively reduces the demand for speed generation modules and greatly saves computing resources. This means that the control frequency of the controller can exceed 100 Hz, fully meeting the real-time requirements of the vehicle driving process. The GPU memory usage is less than 1 GB during runtime, and the trained network has a low usage rate on the GPU. In actual deployment, you may consider replacing it with Jetson Orin Nano 4 GB, which can not only meet performance requirements but also effectively reduce costs.

To verify the effectiveness of this controller, human trajectory data Ding [5] (released by Honda North America Research Institute) is used, covering 37 driving scenarios and a total of 104 h of real driving data. Since the input of the SACPP controller requires speed and acceleration ranges, we use the speed and acceleration data of human drivers to replace the speed and acceleration ranges generated based on the current state of the vehicle, ensuring that the speed produced by SACPP is closer to the actual driving speed of human drivers. We define the improved motor output efficiency rate as $\eta_{I}$ given in (12). By combining vehicle dynamics with the motor efficiency map, the energy consumption rate $R_{ec}$ can be calculated using Equation (13). In these two formulas, $\eta_{SACPP}$, $E_{SACPP}$ represent the average energy efficiency and motor output energy of SACPP, respectively, while $\eta_{h}$, $E_{h}$ represent the average energy efficiency and motor output energy of human drivers.

(12) $$\eta_{I}=\left(\frac{\eta_{SACPP}-\eta_{h}}{\eta_{h}}\right)\times 100\%$$

(13) $$R_{ec}=\left(\frac{E_{h}-E_{SACPP}}{E_{h}}\right)\times 100\%$$

**Figure 13:**
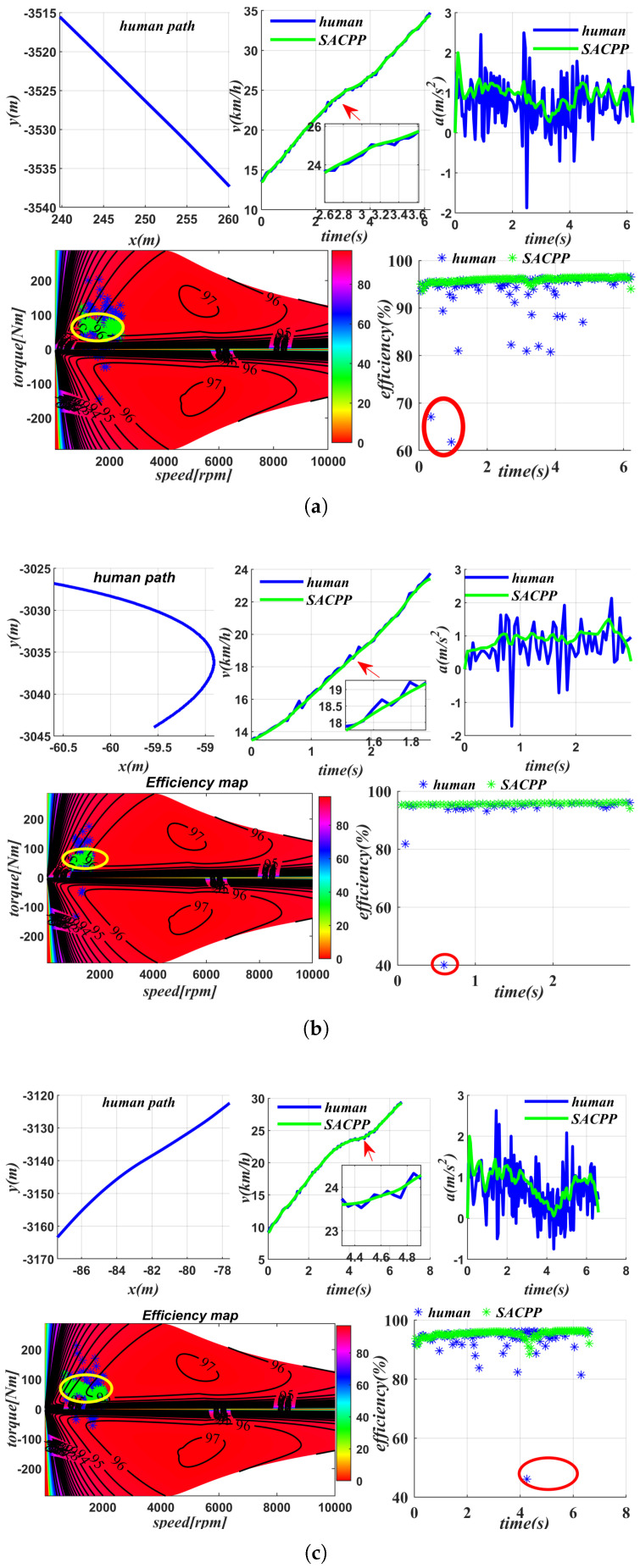
Energy-saving analysis in several driving scenarios: comparison of human driver and SACPP controller performance indicators, including speed *v*, acceleration *a*, and motor energy efficiency map and output efficiency: (**a**) normal straight (**b**) right turn (**c**) intersection.

Figure 13 shows the energy saving result with a normal driving scenario of a human driver, where the driving path of the human driver, *v*, *a*, the motor output efficiency map, and the efficiency details are demonstrated. The subfigure *v* includes the speed of the human driver during driving and the speed finally generated by the proposed SACPP controller based on the human driver as the reference speed. The speed generated by SACPP is similar to the speed of the human driver but smoother. The subfigure *a* compares the acceleration generated by the human driver with the acceleration generated by SACPP, and the results show that the acceleration variation of SACPP is smaller. For the normal straight case shown in Figure 13a, the output efficiency corresponding to SACPP is more concentrated, while the output efficiency of the human driver is more dispersed, and there are some output efficiency points falling by 70%. The average motor output efficiency of human drivers is 92.73%, while that of SACPP is 93.65%, an increase of 0.92%, close to 1%. Higher output efficiency means less energy consumption. In addition, we analyzed scenarios such as avoidance, left turn, and right turn. In these scenarios, due to the low speed and large acceleration and deceleration, the motor output efficiency is reduced. In comparison, the algorithm proposed in this paper performs better in these scenarios, and the actual output efficiency can be improved by up to 1.8%. In the left-turn scenario in Figure 13b, $\eta_{I}$ is 1.23% higher than that of humans, and it exceeds 90%. Additionally, because the generated speed and acceleration are smoother, the overall energy consumption is reduced by 4.5% compared to that of humans, according to the formula. In the intersection scenario shown in Figure 13c, the output efficiency of the motor is 1.13% higher than that of human drivers. As shown in the figure, there are instances where the output efficiency of human drivers is less than 50%, indicating that half of the energy is wasted at certain moments. Although the algorithm proposed in this paper increases the motor’s output efficiency by 1.13%, the overall energy savings exceed 4.5% due to the smoothing of speed and acceleration, according to the formula.

### 6.5. Testing in the Real Vehicle

**Figure 14:**
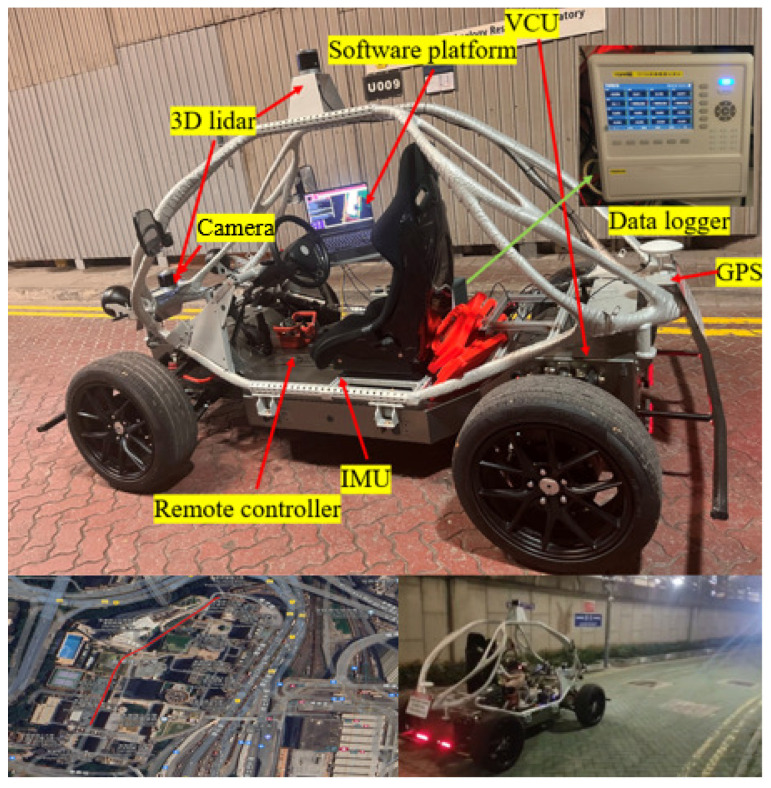
Vehicle testing on an open path on campus.

**Figure 15:**
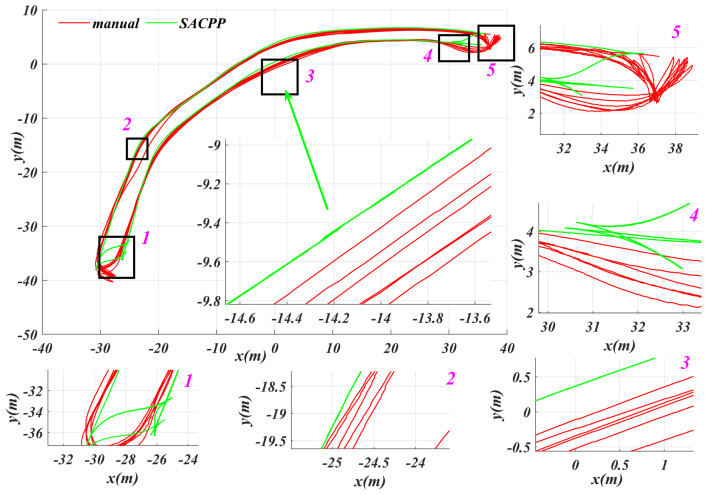
Path tracking performance of the SACPP controller in multiple tests: the human driver drove back and forth on an uneven road at a speed not exceeding 10 km/h, while the vehicle, under the control of our proposed SACPP controller, operated autonomously.

In Figure 14, we selected an open path on campus, where the set path tracking speed must not exceed 10 km/h, and the road is unpaved. Considering the short test area, we adopted a continuous round-trip test method. First, we set the driving path, and the human driver drove the vehicle back and forth along the path, recording the position, speed, and acceleration during the driving process, while using a data logger to save the real-time current of the motor. After the human driver completed the test, we used SACPP as the path-tracking controller and took the human driver’s reference speed as input to record the vehicle’s state and motor current. As seen in Figure 15, when tracking the same path multiple times, the SACPP controller keeps the vehicle close to the reference path and no obvious deviation occurs during the multiple round trips.

**Figure 16:**
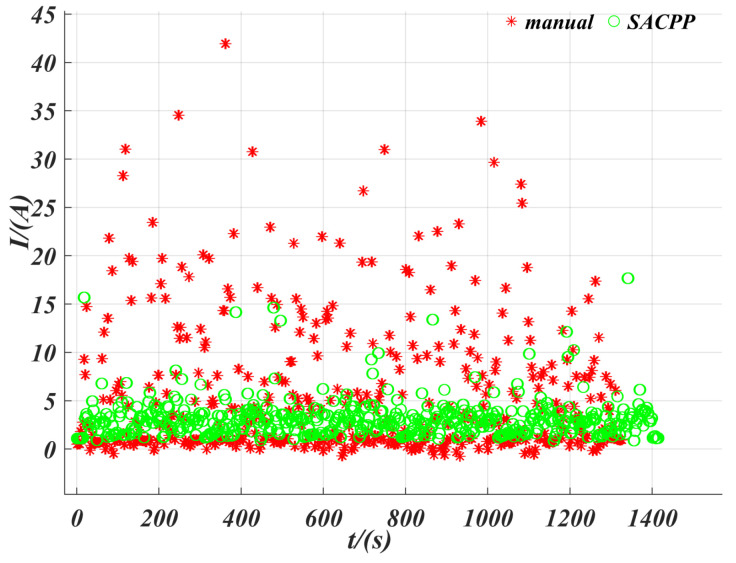
Comparison of the current demand between the SACPP controller and the human driver: The SACPP exhibited smaller and more uniform current fluctuations during path tracking, showing lower energy consumption and higher energy efficiency, ensuring the safety and service life of the motor.

Figure 16 shows a comparison of motor currents between a human driver and a SACPP controller during driving. It can be seen that the current fluctuations required by the SACPP controller are smaller and more uniform, and its current demand is lower than that of a human driver. For the motor, a smaller current means lower energy consumption at the same input voltage. This indicates that the SACPP controller can accomplish the same task with reduced energy consumption. Furthermore, the lateral deviation during continuous path tracking remains within a safe range, which not only improves energy efficiency but also ensures the safety of path tracking. As a result, this contributes to higher motor output efficiency, extends the motor’s service life, and enhances the efficiency of autonomous driving path tracking.

## 7. Conclusions

This paper proposes an accurate path-tracking method for autonomous driving based on U-Net semantic segmentation and a reinforcement learning controller. First, U-Net is used to perform semantic segmentation on the road surface to obtain different types of road surface information. Then, combined with the adaptive capabilities of reinforcement learning, the system can adapt to changing road conditions. At the same time, the motor energy efficiency map is introduced to optimize the motor output, ensuring that it operates above the set threshold, thereby saving energy and extending the motor life. To reduce the computational load, we use a pure-pursuit controller based on a geometric algorithm and employ the adaptive look-ahead nodes generated by reinforcement learning to improve efficiency while ensuring tracking accuracy. Test results show that the algorithm has good anti-interference ability to external and internal disturbances, with the maximum controller time consumption being less than 0.01 s, and the real-time control frequency exceeding 100 Hz, meeting the real-time requirements. The actual machine verification results show that the algorithm performs well in energy saving, tracking accuracy, and motor energy efficiency output. This work aims to improve safety and driving efficiency. First, safety has been improved by eliminating human operation errors through precise control, which effectively solves the problem of frequent accidents at intersections. Second, the reduction in vehicle spacing increases the capacity of the road, thereby improving driving efficiency. Third, the smooth driving trajectory reduces sudden acceleration and deceleration, optimizing energy consumption.

Our future work plans are as follows:

1. We will focus on terrain changes (such as mountain roads and different slopes) to further improve the tracking performance of the controller under various terrains.
2. We will study the impact of different rewards on the stability of the overall controller, and related experiments will be included in subsequent work.
3. We plan to conduct robustness experiments on the proposed algorithm under different weather conditions in future work. However, due to the difficulties in obtaining relevant data and the challenges associated with actual scene testing, this work is currently difficult to implement. Therefore, we will focus on testing the performance of the algorithm in various weather conditions and on steep slope scenarios.
4. Subsequent work will integrate various traffic signs and trajectory predictions of surrounding vehicles to generate a driving speed that takes into account traffic information and surrounding vehicle conditions. This will not only help save energy but also improve travel efficiency. In addition, the algorithm can be deployed on multiple vehicles to achieve collaborative path tracking, further enhancing overall travel efficiency.

## Figures and Tables

**Table 1:** Configuration of the U-Net.

No	Operational Layer	Output	Connected
1	input_layer (InputLayer)	256, 256, 3	
2	conv2d (Conv2D)	256, 256, 16	No. 1
3	max_pooling2d (MaxPooling2D)	128, 128, 16	No. 2
4	conv2d_1 (Conv2D)	128, 128, 32	No. 3
5	max_pooling2d_1 (MaxPooling2D)	64, 64, 32	No. 4
6	conv2_2 (Conv2D)	64, 64, 64	No. 5
7	max_pooling2d_2 (MaxPooling2D)	32, 32, 64	No. 6
8	conv2_3 (Conv2D)	32, 32, 128	No. 7
9	max_pooling2d_3 (MaxPooling2D)	16, 16, 128	No. 8
10	conv2_4 (Conv2D)	16, 16, 256	No. 9
11	conv2d_transpose (Conv2DTranspose)	32, 32, 128	No. 10
12	concatenate (Concatenate)	32, 32, 256	No. 11, 8
13	Conv2_5 (Conv2D)	32, 32, 128	No. 12
14	conv2d_transpose_1 (Conv2DTranspose)	64, 64, 64	No. 13
15	concatenate_1 (Concatenate)	64, 64, 128	No. 14, 6
16	conv2_6 (Conv2D)	64, 64, 64	No. 15
17	conv2d_transpose_2 (Conv2DTranspose)	128, 128, 32	No. 16
18	concatenate_2 (Concatenate)	128, 128, 64	No. 17, 4
19	conv2d_7 (Conv2D)	128, 128, 32	No. 18
20	conv2_9 (Conv2D)	256, 256, 13	No. 19

**Table 2:** SAC hyperparameter settings.

Hyperparameter	Value
Learning rate: actor	0.0003
Learning rate: critic	0.0003
Learning rate: entropy regularization coefficient	0.0003
Discount factor	0.99
Target network update rate	0.005
Replay buffer	1,000,000
Entropy regularization coefficient	0.2

**Table 3:** Parameters of the vehicle.

Parameter	Value
Vehicle mass (kg)	600
Wheel radius (m)	0.3125
Wheel inertia (km/m^2^)	0.25
Battery normal voltage (V)	72
Motor max power (kW)	84
Motor max torque (Nm)	280

**Table 4:** cte results for uncertainties.

Uncertainty Parameters		Max cte (m)	Mean cte (m)
Mass	m	0.220	0.099
	$\frac{9}{10}$ m	0.2548	0.108
	$\frac{11}{10}$ m	0.3025	0.1235
Road surface	Water Puddle	0.5249	0.2207
	Unpaved	0.2549	0.1089
	Paved	0.2321	0.092

## Data Availability

The data that support the findings of this study are available from the corresponding author upon reasonable request.
